# Genetically predicted circulating levels of glycine, glutamate, and serotonin in relation to the risks of three major neurodegenerative diseases: A Mendelian randomization analysis

**DOI:** 10.3389/fnagi.2022.938408

**Published:** 2022-09-07

**Authors:** Ruizhuo Li, Mengjuan Deng, Yuhong Lin, Wenjing Gao, Bohao Liu, Huimin Xia

**Affiliations:** ^1^School of Medicine, South China University of Technology, Guangzhou, China; ^2^Department of Pediatric Surgery, Guangzhou Women and Children’s Medical Center, Guangdong Provincial Clinical Research Center for Child Health, Provincial Key Laboratory of Research in Structure Birth Defect Disease, Guangzhou, China; ^3^Department of Anesthesiology, The Eighth Affiliated Hospital, Sun Yat-sen University, Shenzhen, China; ^4^Zhongshan School of Medicine, The Fifth Affiliated Hospital, Sun Yat-sen University, Guangzhou, China; ^5^Xiangya School of Medicine, The Second Xiangya Hospital, Central South University, Changsha, China

**Keywords:** Alzheimer’s disease, Parkinson’s disease, amyotrophic lateral sclerosis, glycine, glutamate, serotonin, Mendelian randomization, causal inference

## Abstract

It has been previously postulated that blood neurotransmitters might affect risks of neurodegenerative diseases. Here, a Mendelian Randomization (MR) study was conducted to explore whether genetically predicted concentrations of glycine, glutamate and serotonin were associated with risks of Alzheimer’s disease (AD), Parkinson’s disease (PD), and amyotrophic lateral sclerosis (ALS). From three genome-wide association studies of European ancestry, single nucleotide polymorphisms strongly associated with glycine, glutamate and serotonin were selected as genetic instrumental variables. Corresponding summary statistics were also obtained from the latest genome-wide association meta-analyses of AD, PD and ALS. The inverse-variance weighted MR and multiple sensitivity analyses were performed to evaluate causal effects of genetically predicted levels of neurotransmitters on risks of neurodegenerative diseases. The statistical significance threshold was set at *P* < 0.0056 using the Bonferroni-correction, while 0.0056 < *P* < 0.05 was considered suggestive evidence for a causal association. There was a causal association of elevated blood glutamate levels with higher AD risks. The odds ratio (OR) of AD was 1.311 [95% confidence interval (CI), 1.087–1.580; *P* = 0.004] per one standard deviation increase in genetically predicted glutamate concentrations. There was suggestive evidence in support of a protective effect of blood serotonin on AD (OR = 0.607; 95% CI, 0.396–0.932; *P* = 0.022). Genetically predicted glycine levels were not associated with the risk of AD (OR = 1.145; 95% CI, 0.939–1.396; *P* = 0.180). Besides, MR analyses indicated no causal roles of three blood neurotransmitters in PD or ALS. In conclusion, the MR study provided evidence supporting the association of elevated blood glutamate levels with higher AD risks and the association of increased blood serotonin levels with lower AD risks. Triangulating evidence across further study designs is still warranted to elucidate the role of blood neurotransmitters in risks of neurodegenerative diseases.

## Introduction

Alzheimer’s disease (AD), Parkinson’s disease (PD), and amyotrophic lateral sclerosis (ALS) are three major neurodegenerative disorders incurring substantial healthcare burdens worldwide. AD is the primary cause of dementia in the elderly and the most prevalent neurodegenerative disease (Scheltens et al., 2021). PD ranks second most common among neurodegenerative diseases and features movement symptoms (Balestrino and Schapira, 2020). ALS is the leading form of motor neuron disease and progressively leads to the inability to walk, speak, swallow and breathe (Brown and Al-Chalabi, 2017). There have been no disease modifying therapies with definitive efficacy for them so far (Dorst et al., 2018; Piton et al., 2018; Armstrong and Okun, 2020). Identification of biomarkers and therapeutic targets would pave the way for early diagnosis, intervention and prevention of neurodegenerative diseases.

Amino acid neurotransmitters have been postulated to play an essential part in the etiopathogenesis of neurodegenerative disorders (Muñoz et al., 2020; Fagiani et al., 2021; Limanaqi et al., 2021). Glutamate is the main excitatory messenger in the central nervous system (CNS), while glycine is an important inhibitory transmitter. Serotonin (5-Hydroxytryptamine) is one of the major monoamines implicated in such key functions as sleep, mood and appetite. Several studies have been conducted to explore serum metabolic biomarkers, especially these amino acid neurotransmitters for neurodegenerative diseases (Blasco et al., 2018; Jimenez-Jimenez et al., 2020; Pena-Bautista et al., 2020; Jia et al., 2021; Nuzzo et al., 2021; Wichit et al., 2021). But observational researches might come to inconclusive, even contradictory findings. For example, one study (Chouraki et al., 2017) explored associations of plasma metabolites with AD and incident dementia in 2,067 participants from the Framingham Offspring Cohort, and found that higher levels of glutamate were associated with greater risks of AD [hazard ratio (HR), 1.45; 95% confidence interval (CI), 1.11–1.88; *P* = 5.86 × 10^–3^]. However, circulating glutamate levels were inversely associated with AD risk (HR = 0.64; 95% CI, 0.51–0.80; *P* = 1.39 × 10^–3^) in a Chinese cohort of 1,440 dementia-free individuals with five years follow-up (Cui et al., 2020). The diagnostic utility of blood glycine and serotonin levels for AD has also been disputed (Pena-Bautista et al., 2020; Gallo et al., 2021; Nuzzo et al., 2021; Whiley et al., 2021). Likewise, it has yet to be determined whether serum neurotransmitters can serve as promising diagnostic biomarkers and potential therapeutic targets of PD (Tong et al., 2015; Jimenez-Jimenez et al., 2020; Molsberry et al., 2020; Jellen et al., 2021; Wichit et al., 2021) and ALS (Andreadou et al., 2008; Dupuis et al., 2010; Cieslarova et al., 2017; Blasco et al., 2018; Jia et al., 2021). Evidence from these observational studies was compromised due to reverse causation, restricted sample size and confounding factors.

Mendelian randomization (MR) has been emergingly utilized to provide complimentary high-quality evidence concerning potential causal relationships between human diseases and traits. Instrumental variables, as initially applied in econometrics, were introduced into the epidemiologic frameworks of causal inference (Greenland, 2000), and genetic variants, particularly single nucleotide polymorphisms (SNPs), were leveraged in the scenario of MR (Smith and Ebrahim, 2003). Genetic instrumental variables determined at gamete formation are constant in later life and the process of independent assortment of alleles renders a natural and ideal randomization as in conventional randomized control trials (Hemani et al., 2018). As a result, MR studies are powerful in circumventing reverse causations and cofounding biases. The last two decades have witnessed considerable advancement in genome-wide association studies (GWASs) of a large number of diseases and molecular traits (Loos, 2020; Jones et al., 2021). We searched up-to-date GWASs of circulating neurotransmitters and neurodegenerative diseases. Here, we employed publicly accessible data to perform a MR study to investigate causal effects of blood levels of glycine, glutamate and serotonin on risks of AD, PD and ALS.

## Materials and methods

The MR study was based on summary-level data from publicly available GWASs. Ethical approval by review board and informed consent from participants had been completed in original studies.

### Data sources

Summary statistics of human blood neurotransmitter metabolites were obtained from GWASs of European ancestry (Supplementary Table 1). The GWAS meta-analysis of glycine had a total sample size of 80,003 participants from the Fenland, EPIC-Norfolk and INTERVAL studies (Wittemans et al., 2019). Glycine levels underwent the natural logarithm and Z-score transformation and association tests adjusted for age, sex and the first four principal components. We obtained 23 glycine-associated SNPs (*P* ≤ 5 × 10^–8^) as genetic instrumental variables for the ensuing MR analyses. Instrumental SNPs of serotonin were retrieved from a GWAS for 486 metabolite concentrations, comprising up to 6,139 adult individuals from the Cooperative Health Research in the Region of Augsburg study and the TwinsUK cohort (Shin et al., 2014). Since the lead SNP-serotonin pair, that is, the lowest association *P*-value (rs2742351, *P* = 3.23 × 10^–7^) failed to reach the standard genome-wide significance signal, we relaxed the threshold as prior studies did (Kwok et al., 2020; Ng and Schooling, 2020; Baumeister et al., 2021), and selected instrumental variants associated with serotonin at suggestive significance (*P* ≤ 5 × 10^–6^). Lotta et al. (2021) performed a genome-wide meta-analysis of 174 circulating metabolites including a dozen of amino acids in 30,977 individuals from the Fenland, EPIC-Norfolk and INTERVAL studies (Lindsay et al., 2019). We selected SNPs strongly associated (*P* ≤ 5 × 10^–6^) with glutamate concentrations which were mostly measured with the targeted Biocrates AbsoluteIDQ p180 platform (Biocrates, Innsbruck, Austria). For these selected SNPs as instrumental variables, the datasets of summary statistics were curated which mainly included chromosomal coordinates, effect alleles and other alleles, effect sizes, standard errors and P values (Supplementary Tables 2–4).

Summary-level data for SNP-associations with three neurodegenerative diseases were retrieved from latest GWASs of European descent. Kunkle et al. (2019) conducted a GWAS meta-analysis of 21,982 cases with AD and 41,944 healthy controls from four consortia, Alzheimer’s Disease Genetics Consortium, Cohorts for Heart and Aging Research in Genomic Epidemiology Consortium, European Alzheimer’s disease Initiative, Genetic and Environmental Risk in AD/Defining Genetics, Polygenic and Environmental Risk for Alzheimer’s disease Consortium. Nalls et al. (2019) performed a meta-analysis of 17 GWAS datasets of PD and summary statistics of 33,674 cases and 449,056 controls were available by the International Parkinson’s Diseases Genomics Consortium. Lastly, we obtained the largest GWAS of ALS incorporating 20,806 cases and 59,804 controls (Nicolas et al., 2018). Other details on cohort demographics, case definitions and consortia contributions have been described in original studies. For instrumental SNPs which were not present in the outcome datasets of AD, PD and ALS, genetic proxies in linkage disequilibrium (*r*^2^ ≥ 0.8) with them were searched and utilized if available. Effect sizes represented changes in risks of neurodegenerative diseases per additional copy of effect alleles and were harmonized in accordance with SNP-neurotransmitter association data (Supplementary Tables 2–4).

### Statistical analysis

The MR analysis was performed using the R software (version 3.6.3, R Foundation for Statistical Computing, Vienna, Austria) and the *TwoSampleMR* (version 0.5.6) package (Hemani et al., 2018). Assuming effects of SNP_*k*_ on circulating levels of glycine, glutamate and serotonin (${\hat{\beta }}_{X_{k}}$, ${\hat{\sigma }}_{X_{k}}$) and on risks of AD, PD and ALS (${\hat{\beta }}_{Y_{k}}$, ${\hat{\sigma }}_{Y_{k}})$ from the GWAS additive model, estimates of causal associations of neurotransmitters with neurodegenerative diseases (SNP*k*→*X*_*k*_→Y*k*) can be given by the Wald method (Walker et al., 2019), effect size


$${\hat{\theta }}_{k}={\hat{\beta }}_{Y_{k}}/{\hat{\beta }}_{X_{k}}$$

and standard error


$$s\,e\,\left({\hat{\theta }}_{k}\right)={\hat{\sigma }}_{Y_{k}}/{\hat{\beta }}_{X_{k}}$$

The inverse variance weighted (IVW) method was primarily employed to combine multiple instrumental SNPs and generate one overall estimate (Burgess et al., 2013), effect size


θ^I⁢V⁢W=Σk⁢β^Xk⁢β^Yk⁢σ^Yk-2/Σk⁢β^Xk 2⁢σ^Yk-2

and standard error


σ^I⁢V⁢W=1/Σk⁢β^Xk 2⁢σ^Yk-2

Notably, the IVW method would be biased when invalid instrumental variables existed. And thus, two complementary means, weighted median and MR-Egger were implemented as sensitivity analyses. The weighted median model was largely robust when more than half of instrumental SNPs were valid (Bowden et al., 2016). The MR-Egger approach was able to examine unbalanced horizontal pleiotropy through its regression intercept, meanwhile the regression slope would give a causal estimate accounting for pleiotropic effects (Burgess and Thompson, 2017). Forest plots were presented to visualize associations of neurotransmitters with neurodegenerative diseases, with odds ratio (OR) and 95% confidence interval (CI) denoted changes in risks of AD, PD or ALS per one-standard deviation (SD) increment in circulating concentrations of glycine, glutamate or serotonin. Besides, the scatter and leave-one-out plots were utilized to examine potential outliers and heterogenous effects. The Bonferroni-adjusted significance threshold was set at *P* < 0.05/(3*3) = 0.0056, whereas 0.0056 < *P* < 0.05 was suggestive of a causal effect.

## Results

### Associations of blood glycine levels with three neurodegenerative diseases

**FIGURE 1:**
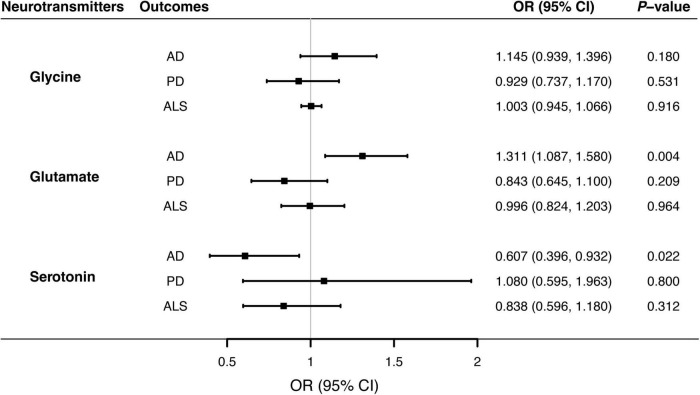
Associations of genetically predicted blood neurotransmitter levels of with risks of neurodegenerative diseases via Mendelian randomization analyses. Causal effects of circulating glycine glutamate, and serotonin concentrations on the risks of AD, PD, and ALS were estimated by the inverse-variance weighted MR, and illustrated with forest plots. Solid blocks denoted effect sizes of point estimates, and horizontal lines represented 95% confidence intervals. Given the Bonferroni-corrected threshold at 0.05/9 (0.0055) to account for multiple testing, the study showed that elevated glutamate levels were associated with higher risks of AD (*P* = 0.004). There was suggestive evidence supporting the protective effect of circulating serotonin on AD (*P* = 0.022). AD, Alzheimer’s disease; ALS, amyotrophic lateral sclerosis; CI; confidence interval; MR, Mendelian randomization; OR, odds ratio; PD, Parkinson’s disease.

Overall, MR analyses suggested that genetically predicted blood concentrations of glycine were not associated with risks of three neurodegenerative diseases. The OR of AD was 1.145 (95% CI, 0.939–1.396; *P* = 0.180) per one SD increase in circulating glycine levels (Figure 1). There was no evidence for the causal effects of genetically predicted glycine concentrations on genetic susceptibilities to PD (OR = 0.929; 95% CI, 0.737–1.170; *P* = 0.531) or ALS (OR = 1.003; 95% CI, 0.945–1.066; *P* = 0.916). No unbalance horizontal pleiotropy was detected by the examination of MR Egger intercepts (Table 1). Although there seemed a little heterogeneity in the MR analysis of glycine on AD and PD, it was not evident by checking the scatter plots (Supplementary Figure 1) and leave-one-out forest plots (Supplementary Figure 2) and might not affect the robustness of MR results.

**TABLE 1:** Mendelian randomization analyses of circulating glycine levels on three neurodegenerative diseases.

Outcomes	SNPs	OR (95% CI)	*P*-value	Cochran’s *Q*	*P*-value	MR-Egger intercept	*P*-value
**AD**							
Inverse-variance weighted	19	1.145 (0.939, 1.396)	0.180	33.030	0.017		
Weighted median	19	1.138 (0.920, 1.407)	0.235				
MR-Egger regression	19	1.101 (0.683, 1.777)	0.697			0.002	0.863
**PD**							
Inverse-variance weighted	23	0.929 (0.737, 1.170)	0.531	34.654	0.042		
Weighted median	23	1.058 (0.784, 1.429)	0.713				
MR-Egger regression	23	0.940 (0.553, 1.598)	0.822			–0.001	0.960
**ALS**							
Inverse-variance weighted	23	1.003 (0.945, 1.066)	0.916	21.103	0.514		
Weighted median	23	1.002 (0.939, 1.070)	0.945				
MR-Egger regression	23	1.004 (0.931, 1.082)	0.921			0.000	0.981

AD, Alzheimer’s disease; ALS, amyotrophic lateral sclerosis; CI, confidence interval; OR, odds ratio; MR, Mendelian randomization; PD, Parkinson’s disease; SNP, single nucleotide polymorphism.

### Associations of blood glutamate levels with three neurodegenerative diseases

Genetically predicted higher glutamate levels were associated with an increased risk of AD (OR = 1.311; 95% CI, 1.087–1.580; *P* = 0.004). But MR analyses did not support causal relationship between blood glutamate concentrations and risks of PD or ALS. The OR of PD was 0.843 (95% CI, 0.645–1.100; *P* = 0.209), and the OR of ALS was 0.996 (95% CI, 0.824–1.203; *P* = 0.964) per one SD increment in genetically proxied glutamate levels. Estimates of causal associations of glutamate with AD given by the weighted median and MR-Egger approaches were directionally consistent (Table 2), albeit not statistically significant. Besides, there was no evidence of evident heterogenous effects or unbalanced horizontal pleiotropy via sensitivity analyses, indicating the causal effect of glutamate on AD was generally robust.

**TABLE 2:** Mendelian randomization analyses of circulating glutamate levels on three neurodegenerative diseases.

Outcomes	SNPs	OR (95% CI)	*P*-value	Cochran’s *Q*	*P*-value	MR-Egger intercept	*P*-value
**AD**							
Inverse-variance weighted	20	1.311 (1.087, 1.580)	0.004	10.195	0.948		
Weighted median	20	1.269 (0.970, 1.660)	0.082				
MR-Egger regression	20	1.190 (0.816, 1.737)	0.378			0.004	0.573
**PD**							
Inverse-variance weighted	21	0.843 (0.645, 1.100)	0.209	15.623	0.740		
Weighted median	21	0.840 (0.575, 1.227)	0.368				
MR-Egger regression	21	0.900 (0.546, 1.485)	0.685			–0.003	0.764
**ALS**							
Inverse-variance weighted	23	0.996 (0.824, 1.203)	0.964	30.911	0.098		
Weighted median	23	1.027 (0.823, 1.282)	0.812				
MR-Egger regression	23	1.631 (1.161, 2.291)	0.010			–0.023	0.004

AD, Alzheimer’s disease; ALS, amyotrophic lateral sclerosis; CI, confidence interval; OR, odds ratio; MR, Mendelian randomization; PD, Parkinson’s disease; SNP, single nucleotide polymorphism.

### Associations of blood serotonin levels with three neurodegenerative diseases

MR analyses suggested that there was a causal association of blood serotonin with AD risk (OR = 0.607; 95% CI, 0.396–0.932; *P* = 0.022), indicating a potentially protective effect for serotonin on AD onset. Nevertheless, there was no evidence in support of causal effects of genetically predicted serotonin levels on the risk of PD (OR = 1.080; 95% CI, 0.595–1.963; *P* = 0.800), and ALS (OR = 0.838; 95% CI, 0.596–1.180; *P* = 0.312). The weighted median estimate supported a causal relationship between serotonin and AD (OR = 0.517; 95% CI, 0.300–0.893; *P* = 0.018), whereas the MR-Egger regression slope had inadequate power to detect the causal effect (OR = 1.010) as indicated by the wide CI (0.222–4.587). Since there was no evidence for significant heterogeneity or pleiotropy (Table 3), the effect size given by the IVW method was considered preferentially. Further inspecting sensitivity analysis results, scatter plots (Supplementary Figure 1) and leave-one-out analyses (Supplementary Figure 2) suggested no existence of outlying SNPs which would drive the causal effect unproportionally. On the whole, the MR analysis provided suggestive evidence for a protective effect of serotonin on AD.

**TABLE 3:** Mendelian randomization analyses of circulating serotonin levels on three neurodegenerative diseases.

Outcomes	SNPs	OR (95% CI)	*P*-value	Cochran’s *Q*	*P*-value	MR-Egger intercept	*P*-value
**AD**							
Inverse-variance weighted	22	0.607 (0.396, 0.932)	0.022	26.983	0.171		
Weighted median	22	0.517 (0.300, 0.893)	0.018				
MR-Egger regression	22	1.010 (0.222, 4.587)	0.990			–0.010	0.500
**PD**							
Inverse-variance weighted	23	1.080 (0.595, 1.963)	0.800	35.575	0.046		
Weighted median	23	2.449 (1.194, 5.024)	0.015				
MR-Egger regression	23	1.025 (0.121, 8.689)	0.982			0.001	0.960
**ALS**							
Inverse-variance weighted	23	0.838 (0.596, 1.180)	0.312	11.571	0.977		
Weighted median	23	0.899 (0.563, 1.434)	0.654				
MR-Egger regression	23	0.472 (0.102, 2.176)	0.346			0.011	0.457

AD, Alzheimer’s disease; ALS, amyotrophic lateral sclerosis; CI, confidence interval; OR, odds ratio; MR, Mendelian randomization; PD, Parkinson’s disease; SNP, single nucleotide polymorphism.

## Discussion

The study demonstrated that genetically predicted blood glutamate and serotonin concentrations were associated with AD risks. Specifically, individuals with increased glutamate levels were more likely predisposed to AD, whereas increased serotonin levels were associated with lower risks of AD. There was no evidence supporting causal relationships between blood levels concentrations of glycine, glutamate or serotonin and onset risks of PD or ALS.

According to the MR analysis, glutamate was one risk factor for AD, which might be partly explained by its excitotoxicity to neurons. Amyloid beta plaques, tau-associated neurofibrillary tangles, microglial activation and neuroinflammation have been established as key pathological changes in AD (Li et al., 2018). Tau pathology might have an influence on glutamatergic neurons, and glutamate in turn would facilitate tau phosphorylation and neuronal damage (Leng and Edison, 2021). Release of glutaminase, the enzyme expediting the generation of glutamate from glutamine, was proposed as another possible mechanism through which activated microglia would kill neurons (Chi et al., 2018). Glutamate could be toxic to depolarized neurons or render neurons sufficiently vulnerable to phagocytosis by microglia (Brown and Vilalta, 2015). The most ideal analysis should come from data of glutamate from cerebrospinal fluid or brain tissue, whereas this study was focusing on the association of blood glutamate levels with genetic liabilities to AD. Admittedly, it remains ever more elusive how circulating glutamate in blood would exert its influence on the etiology and pathogenesis of AD. However, previous studies have proposed consistent findings or plausible explanations. Reduction of blood glutamate levels by inducing autoantibodies to glutamate (Trekova et al., 2002; Davydova et al., 2007) was reported to lower brain glutamate levels and improve memory and behavioral performance, presumptively, by decreasing neuronal damages. Likewise, brain glutamate levels could be indirectly decreased by reducing blood glutamate levels using branched chain alpha-keto acids (Polis and Samson, 2020) or glutamate-pyruvate transaminase enzymes (Boyko et al., 2012). Besides, peripheral concentrations of glutamate in the blood seemed to be more proportional or similar to tissue concentrations in the hippocampus, given that the blood-brain barrier is not as well-developed in the hippocampus that other brain regions (Miulli et al., 1993). A good correlation between glutamate levels in the blood and cerebrospinal fluid was reported similarly, and blood glutamate was proposed as a promising assay for early screening of AD (Jimenez-Jimenez et al., 1998; Chang et al., 2021; Nuzzo et al., 2021), considering that the measurement of glutamate from blood samples could be less invasive, less expensive and more practical that from cerebrospinal fluids or brain tissues. In this regard, the study provided additional evidence supporting the association of blood levels of glutamate with risks of AD, but the specific mechanism underlying the association warrants further exploration.

Promising diagnostic utility of serotonin, together with other monoamines and their derivatives, for the early diagnosis of AD has also aroused great interest (Gallo et al., 2021). In a study based on the European-wide AddNeuroMed/Dementia Case Register biobank repositories (n = 354), a decreasing trend in serum concentrations of serotonin was reported in line with clinical classification, namely the elderly control, mild cognitive impairment and AD (Whiley et al., 2021). But in a Spanish cohort (*n* = 50) there was no statistical difference (*P* = 0.372) in plasma serotonin levels between mild cognitive impairment–AD and controls (Pena-Bautista et al., 2020). Researchers have tried to employ in vitro and in vivo models to elucidate the role of serotonin in AD as well. One surface plasmon resonance study demonstrated that serotonin could facilitate monomeric Aβ binding to serum albumin, which was hypothesized to be a promising way to prevent the onset and progression of AD (Litus et al., 2021). Morgese et al. (2021) reported that serotonin levels were decreased in Aβ-injected rats which was established as a rat model mimicking the early phase of AD. In another recent experiment (Sivasaravanaparan et al., 2022), nevertheless, chronic treatment with paroxetine—a selective serotonin reuptake inhibitor, failed to mitigate Aβ pathology and microgliosis in APPSWE/PS1ΔE9 mice. In addition, the serotonin transporter (*SLC6A4*) which regulates the serotonin concentrations and serves as the target of selective serotonin reuptake inhibitors, might be associated with AD risks (Polito et al., 2011; Yamazaki et al., 2016). For example, in a meta-analysis (Yamazaki et al., 2016) the L/L genotype in the serotonin transporter length polymorphic region significantly reduced the risk of AD in Europeans and the L alleles were previously reported to associate with lower *SLC6A4* expressions in peripheral leukocytes (Hu et al., 2006). Presumably, there might be protective effects of decreased expressions of the serotonin transporter and increased concentrations of serotonin in lowering AD risks. Consistently, our MR analysis indicated a protective effect of serotonin, that is, elevated blood serotonin levels were associated with lower AD risks. Serotonergic deficits in extensive brain regions were proposed to explain the irritability and agitation in AD patients (Chakraborty et al., 2019). It is true that the serotonergic system alteration has been tied to behavioral, cognitive, and psychiatric presentations. The complex changes in serotonin, serotonin transporters and serotonin receptors underlying the onset and progression of AD, however, warrant further clarification. Notably, we did not explore the possible effect of AD on serotonin using a reverse MR yet, while several studies also reported that amyloid-β accumulations were associated with altering serotonin levels (Wang et al., 2018; Tin et al., 2019). Overall, the clinical and translational exploration of the potential role of serotonin in AD still has a long way to go.

In the MR study, there was no evidence for the effects of blood glycine levels on genetic liabilities to AD, and whether there existed causal relationships between three blood neurotransmitters and genetic susceptibilities to PD and ALS was unclear. Prior studies explored the association of glycine with ALS, and glycine appeared to be a potential diagnostic biomarker (Jia et al., 2021) or an indicator of progression (Blasco et al., 2018). Serotonin seemed to manifest certain utility in the predictive and therapeutic aspects of ALS (Sandyk, 2006; Dupuis et al., 2010). Glutamate excitotoxicity as one major breakthrough in neurobiology, has also been linked with the pathogenesis of ALS (Holecek and Rokyta, 2018). In comparison with healthy individuals (n = 20), increased plasma glutamate concentrations measured by high-pressure liquid chromatography were identified in ALS patients (n = 65), which also correlated with longer duration (Andreadou et al., 2008). The N-methyl-D-aspartate receptor, one of three major types of ionotropic glutamate receptors, requires the primary agonist, glutamate and the co-agonist, glycine or D- enantiomer of serine to be activated, and its modulation is thought to be critical in mediating synaptic plasticity, promoting neuronal survival and preventing neurodegeneration (Kalia et al., 2008; Piubelli et al., 2021). However, findings from our MR analysis failed to confirm the association of glutamate with ALS. In this regard, alterations in other components involved in glutamate receptor signaling rather than glutamate itself might play an essential part in ALS. To validate the role of neurotransmitters in neurodegenerative diseases *in vivo* and unveil the specific mechanism, nonetheless, more basic researches have yet to do.

Several limitations should be acknowledged in the study. Firstly, GWAS datasets utilized in the MR analyses were sourced from cohorts of European descent. Hence, great cautions should be exercised when generalizing the findings to other populations. Secondly, neurotransmitters might play an essential part in neurodegeneration, and circulating levels of neurotransmitters in cerebrospinal fluid would be more closely related to risks of neurodegenerative diseases than those in blood. Nevertheless, large-scale proteomic or metabolomic association statistics of neurotransmitters in the cerebrospinal fluid, let alone in brain tissues, were hitherto unavailable. Lastly, although other key neurotransmitters like acetylcholine, gamma-aminobutyric acid and dopamine have been implicated in neurodegenerative diseases, we could not perform MR analyses to explore their causal effects in the present study. Once corresponding genome-wide association data are available, the MR framework can be exploited to comprehensively investigate the causal roles of blood, cerebrospinal, and intracellular neurotransmitters from region specific brain tissues, further benefiting the establishment of promising diagnostic biomarkers and interventional targets for neurodegenerative disease.

In conclusion, the MR study provided evidence supporting the association of elevated blood glutamate levels with higher AD risks and the association of increased blood serotonin levels with lower AD risks. Triangulating evidence across further study designs is still warranted to validate the detrimental role of glutamate and the protective role of serotonin in genetic susceptibilities to AD.

## Data availability statement

The original contributions presented in this study are included in the article/Supplementary material, further inquiries can be directed to the corresponding authors.

## Author contributions

RL, BL, and HX contributed to conceptualization of the work. RL, MD, and WG performed the main analysis. RL, MD, and YL drafted and reviewed the main manuscript. BL and HX contributed to the project supervision. All authors contributed to the article and approved the final version of the manuscript.
