# Supplementary material for: Genetically predicted circulating levels of glycine, glutamate, and serotonin in relation to the risks of three major neurodegenerative diseases: A Mendelian randomization analysis
Source: Front Aging Neurosci. 2022 Sep 7;14:938408. doi: 10.3389/fnagi.2022.938408 (PMC9490425; doi:10.3389/fnagi.2022.938408)
Supplement: Supplementary file 1 [file Data_Sheet_1.PDF]

Supplementary Materials

Supplementary Table 1. Source datasets in the Mendelian randomization study

Supplementary Table 2. Summary-level data associated with blood neurotransmitters and Alzheimer's disease

Supplementary Table 3. Summary-level data associated with blood neurotransmitters and Parkinson’s disease

Supplementary Table 4. Summary-level data associated with blood neurotransmitters and amyotrophic lateral sclerosis

Supplementary Figure 1. Scatter plots in Mendelian randomization analyses of circulating glycine, glutamate and serotonin on three neurodegenerative diseases

Supplementary Figure 2. Leave-one-out plots in Mendelian randomization analyses of circulating glycine, glutamate and serotonin on three neurodegenerative diseases

Supplementary Table 1. Source datasets in the Mendelian randomization study

Trait	Sample Size	Cases	Controls	Ancestry	Author	PMID	Data access Link
Glycine	80,003	–	–	European	Wittemans et al.	30837465	https://www.ebi.ac.uk/gwas/studies/GCST007638
Glutamate	30,977	–	–	European	Lotta et al.	33414548	https://omicscience.org/apps/crossplatform/
Serotonin	5,791	–	–	European	Shin *et al.*	24816252	http://metabolomics.helmholtz-muenchen.de/gwas/
AD	63,926	21,982	41,944	European	Kunkle *et al.*	30820047	https://www.niagads.org/datasets/ng00075
PD	482,730	33,674	449,056	European	Nalls *et al.*	31701892	https://pdgenetics.org/resources
ALS	80,610	20,806	59,804	European	Nicolas *et al.*	29566793	http://als.umassmed.edu/

Abbreviations: AD, Alzheimer's disease; ALS, amyotrophic lateral sclerosis; PD, Parkinson’s disease; SNP, single nucleotide polymorphism.

Supplementary Table 2. Summary-level data associated with blood neurotransmitters and Alzheimer's disease

Exposures	SNP	Position	EA/OA	Association with neurotransmitters			Association with Alzheimer's disease		
				Beta	SE	*P*-value	Beta	SE	*P*-value
Glycine	rs9987289	8:9183358	A/G	0.124	0.01	1.74×10^{-49}	0.027	0.026	0.292
Glycine	rs9923732	16:81110903	A/G	0.119	0.011	1.22×10^{-41}	0.010	0.027	0.717
Glycine	rs17591030	9:6550024	C/T	0.08	0.006	1.88×10^{-40}	0.031	0.017	0.068
Glycine	rs4947534	7:56079094	C/T	0.072	0.007	7.12×10^{-34}	0.009	0.016	0.602
Glycine	rs9862438	3:125910381	T/C	0.058	0.006	1.13×10^{-30}	-0.014	0.014	0.344
Glycine	rs4646961	1:76217169	A/G	0.048	0.006	8.41×10^{-19}	-0.036	0.016	0.021
Glycine	rs676996	9:136146077	T/G	0.04	0.006	4.39×10^{-15}	0.005	0.015	0.764
Glycine	rs2545801	5:176841339	C/T	0.042	0.007	7.23×10^{-14}	-0.010	0.018	0.565
Glycine	rs12297321	12:47109387	T/C	0.048	0.008	7.41×10^{-13}	0.016	0.020	0.425
Glycine	rs10740134	10:65315433	T/C	0.038	0.006	1.18×10^{-12}	0.011	0.014	0.439
Glycine	rs8078686	17:45735706	C/T	0.035	0.006	3.66×10^{-11}	0.051	0.014	0.000
Glycine	rs543159	6:160776017	A/C	0.035	0.006	4.20×10^{-10}	0.030	0.014	0.037
Glycine	rs71640034	4:187161048	A/G	0.034	0.006	5.57×10^{-10}	-0.007	0.015	0.664
Glycine	rs10184004	2:165508389	T/C	0.036	0.006	1.53×10^{-9}	-0.018	0.014	0.204
Glycine	rs2280195	15:58467095	A/G	0.028	0.006	3.15×10^{-9}	0.020	0.014	0.164
Glycine	rs273510	19:18223350	A/G	0.034	0.006	3.57×10^{-9}	-0.004	0.016	0.820
Glycine	rs201393666	15:43685807	A/C	0.097	0.017	2.64×10^{-8}	0.030	0.039	0.434
Glycine	rs3105793	5:90226061	A/G	0.028	0.006	4.04×10^{-8}	0.019	0.016	0.255
Glycine	rs156380	5:53378450	C/T	0.031	0.007	4.50×10^{-8}	-0.017	0.018	0.335
Glutamate	rs7979478	12:121423376	A/G	-0.018	0.014	1.52×10^{-10}	-0.015	0.015	0.319
Glutamate	rs1183910	12:121420807	G/A	0.016	0.015	8.58×10^{-9}	0.007	0.015	0.638
Glutamate	rs1169312	12:121441461	G/T	0.008	0.014	2.37×10^{-7}	0.013	0.015	0.381
Glutamate	rs10842676	12:26261394	A/G	-0.04	0.014	2.91×10^{-7}	-0.006	0.015	0.697
Glutamate	rs41356552	15:60957248	A/G	0.056	0.017	3.59×10^{-7}	0.016	0.018	0.365
Glutamate	rs1182933	12:121454622	C/T	0.011	0.015	5.94×10^{-7}	0.007	0.016	0.661
Glutamate	rs2650000	12:121388962	A/C	-0.004	0.015	1.52×10^{-6}	-0.009	0.015	0.546
Glutamate	rs7110183	11:72551035	C/T	0.061	0.017	1.54×10^{-6}	0.006	0.018	0.731

Glutamate	rs5760492	22:24995202	G/A	-0.023	0.015	1.57×10^{-6}	0.003	0.016	0.835
Glutamate	rs10805985	6:74606851	G/A	0.046	0.015	2.09×10^{-6}	0.032	0.016	0.046
Glutamate	rs12301299	12:991710	T/C	-0.034	0.019	2.12×10^{-6}	0.014	0.020	0.477
Glutamate	rs10774579	12:121405210	T/C	-0.021	0.014	2.30×10^{-6}	-0.014	0.014	0.340
Glutamate	rs4895778	6:149436652	G/A	0.041	0.018	2.40×10^{-6}	0.001	0.018	0.973
Glutamate	rs12591786	15:60902512	C/T	0.048	0.02	2.66×10^{-6}	0.041	0.020	0.042
Glutamate	rs3816411	3:13677751	T/C	-0.036	0.015	3.58×10^{-6}	0.012	0.015	0.441
Glutamate	rs7956891	12:3475998	G/A	0.023	0.017	3.99×10^{-6}	0.000	0.019	0.983
Glutamate	rs3775330	4:30728284	A/G	0.064	0.021	4.24×10^{-6}	0.012	0.021	0.565
Glutamate	rs10041854	5:141464898	C/A	0.077	0.023	4.44×10^{-6}	0.019	0.022	0.401
Glutamate	rs880626	15:60983429	G/A	0.049	0.017	4.55×10^{-6}	0.014	0.016	0.385
Glutamate	rs10747028	9:80813104	C/A	0.053	0.014	4.81×10^{-6}	0.023	0.014	0.101
Serotonin	rs2742351	2:179541899	C/G	-0.024	0.005	3.23×10^{-7}	-0.012	0.016	0.450
Serotonin	rs10516056	5:168468537	T/C	-0.022	0.005	6.99×10^{-7}	-0.001	0.019	0.958
Serotonin	rs5764174	22:44033934	T/C	-0.019	0.004	9.61×10^{-7}	-0.016	0.017	0.335
Serotonin	rs8113773	19:30350139	A/G	0.016	0.003	1.12×10^{-6}	-0.045	0.015	0.003
Serotonin	rs7868774	9:138016943	A/G	-0.019	0.004	1.78×10^{-6}	0.014	0.016	0.401
Serotonin	rs12646122	4:156246202	T/C	-0.05	0.01	1.80×10^{-6}	0.060	0.053	0.260
Serotonin	rs9614255	22:44056241	A/G	-0.019	0.004	1.93×10^{-6}	-0.015	0.017	0.378
Serotonin	rs825278	2:222561267	T/C	-0.091	0.019	2.06×10^{-6}	0.035	0.060	0.564
Serotonin	rs5763947	22:44035303	A/G	-0.019	0.004	2.12×10^{-6}	-0.016	0.017	0.341
Serotonin	rs11103693	9:138016657	T/C	-0.019	0.004	3.09×10^{-6}	0.014	0.016	0.401
Serotonin	rs11103694	9:138016676	T/C	-0.019	0.004	3.10×10^{-6}	0.015	0.016	0.370
Serotonin	rs34724	19:30369010	T/G	-0.015	0.003	3.20×10^{-6}	0.033	0.015	0.027
Serotonin	rs737788	22:44039285	T/C	-0.018	0.004	3.62×10^{-6}	-0.018	0.017	0.287
Serotonin	rs3753556	1:175162723	T/C	0.015	0.003	3.66×10^{-6}	-0.012	0.014	0.413
Serotonin	rs10881427	1:107172665	T/G	-0.015	0.003	3.69×10^{-6}	-0.006	0.014	0.658
Serotonin	rs9614170	22:44061009	T/G	-0.018	0.004	3.87×10^{-6}	-0.015	0.017	0.367
Serotonin	rs10953038	7:90857574	T/C	0.022	0.005	4.12×10^{-6}	-0.002	0.020	0.924
Serotonin	rs6466265	7:78160248	A/G	0.019	0.004	4.15×10^{-6}	-0.038	0.018	0.032
Serotonin	rs34722	19:30366802	A/C	0.015	0.003	4.36×10^{-6}	-0.020	0.015	0.188
Serotonin	rs34720	19:30366245	A/G	0.015	0.003	4.40×10^{-6}	-0.020	0.015	0.194

Serotonin	rs34725	19:30370237	A/G	-0.018	0.004	4.62×10^{-6}	0.019	0.016	0.217
Serotonin	rs17039706	9:138016002	T/C	-0.019	0.004	4.74×10^{-6}	0.012	0.017	0.472

Note: For genetic variants (rs201393666, rs7979478, rs3753556) not available in the summary statistics of AD, proxied SNPs in linkage disequilibrium with them were utilized as bellows, rs149181595 ($r^2 = 1.00$), rs7970695 ($r^2 = 0.99$), rs3753555 ($r^2 = 1.00$), respectively.

Abbreviations: EA, effect allele; OA, other allele; SNP, single nucleotide polymorphism.

Supplementary Table 3. Summary-level data associated with blood neurotransmitters and Parkinson's disease

Exposures	SNP	Position	EA/OA	Association with neurotransmitters			Association with Parkinson's disease		
				Beta	SE	*P*-value	Beta	SE	*P*-value
Glycine	rs9987289	8:9183358	A/G	0.124	0.01	1.74×10^{-49}	-0.062	0.031	0.047
Glycine	rs9923732	16:81110903	A/G	0.119	0.011	1.22×10^{-41}	0.011	0.032	0.729
Glycine	rs17591030	9:6550024	C/T	0.08	0.006	1.88×10^{-40}	-0.063	0.026	0.013
Glycine	rs4947534	7:56079744	C/T	0.072	0.007	7.12×10^{-34}	0.047	0.022	0.035
Glycine	rs9862438	3:125910381	T/C	0.058	0.006	1.13×10^{-30}	0.018	0.023	0.426
Glycine	rs4646961	1:76217169	A/G	0.048	0.006	8.41×10^{-19}	0.014	0.019	0.442
Glycine	rs2545801	5:176841339	C/T	0.042	0.007	7.23×10^{-14}	-0.042	0.022	0.058
Glycine	rs12297321	12:47109387	T/C	0.048	0.008	7.41×10^{-13}	0.022	0.031	0.469
Glycine	rs10740134	10:65315433	T/C	0.038	0.006	1.18×10^{-12}	-0.014	0.017	0.414
Glycine	rs8078686	17:45735706	C/T	0.035	0.006	3.66×10^{-11}	0.024	0.017	0.164
Glycine	rs148685782	4:155533035	G/C	0.309	0.049	2.01×10^{-10}	0.292	0.205	0.154
Glycine	rs543159	6:160776017	A/C	0.035	0.006	4.20×10^{-10}	-0.007	0.018	0.690
Glycine	rs71640034	4:187174683	A/G	0.034	0.006	5.57×10^{-10}	-0.011	0.017	0.511
Glycine	rs10900807	5:131757480	G/C	0.036	0.007	1.26×10^{-9}	0.002	0.021	0.920
Glycine	rs10184004	2:165508389	T/C	0.036	0.006	1.53×10^{-9}	0.002	0.017	0.913
Glycine	rs2280195	15:58467095	A/G	0.028	0.006	3.15×10^{-9}	0.007	0.022	0.761
Glycine	rs273510	19:18223350	A/G	0.034	0.006	3.57×10^{-9}	-0.013	0.019	0.511
Glycine	rs190595610	10:32274880	A/G	0.253	0.056	8.96×10^{-9}	-0.085	0.285	0.767
Glycine	rs2638314	12:56866334	A/T	0.042	0.007	1.52×10^{-8}	-0.025	0.024	0.299
Glycine	rs201393666	15:43757184	A/C	0.097	0.017	2.64×10^{-8}	0.006	0.051	0.904
Glycine	rs9514191	13:104520138	C/G	0.034	0.006	3.10×10^{-8}	0.030	0.024	0.212
Glycine	rs3105793	5:90226061	A/G	0.028	0.006	4.04×10^{-8}	0.001	0.021	0.970
Glycine	rs156380	5:53378450	C/T	0.031	0.007	4.50×10^{-8}	-0.072	0.028	0.009
Glutamate	rs7979478	12:121423376	A/G	-0.018	0.014	1.52×10^{-10}	0.008	0.017	0.630
Glutamate	rs1183910	12:121420807	G/A	0.016	0.015	8.58×10^{-9}	-0.007	0.018	0.709
Glutamate	rs1169312	12:121441461	G/T	0.008	0.014	2.37×10^{-7}	0.006	0.018	0.726
Glutamate	rs10842676	12:26261394	A/G	-0.04	0.014	2.91×10^{-7}	0.017	0.023	0.449

Glutamate	rs41356552	15:60957248	A/G	0.056	0.017	3.59×10^{-7}	-0.011	0.029	0.695
Glutamate	rs1182933	12:121454622	C/T	0.011	0.015	5.94×10^{-7}	0.007	0.018	0.717
Glutamate	rs2650000	12:121388962	A/C	-0.004	0.015	1.52×10^{-6}	0.010	0.018	0.576
Glutamate	rs7110183	11:72551035	C/T	0.061	0.017	1.54×10^{-6}	-0.019	0.028	0.480
Glutamate	rs5760492	22:24995202	G/A	-0.023	0.015	1.57×10^{-6}	0.014	0.018	0.418
Glutamate	rs10805985	6:74606851	G/A	0.046	0.015	2.09×10^{-6}	0.035	0.021	0.104
Glutamate	rs12301299	12:991710	T/C	-0.034	0.019	2.12×10^{-6}	0.005	0.029	0.866
Glutamate	rs10774579	12:121405210	T/C	-0.021	0.014	2.30×10^{-6}	-0.003	0.017	0.848
Glutamate	rs4895778	6:149436652	G/A	0.041	0.018	2.40×10^{-6}	-0.037	0.027	0.181
Glutamate	rs12591786	15:60902512	C/T	0.048	0.02	2.66×10^{-6}	0.005	0.027	0.846
Glutamate	rs3816411	3:13677751	T/C	-0.036	0.015	3.58×10^{-6}	0.050	0.023	0.031
Glutamate	rs7956891	12:3475998	G/A	0.023	0.017	3.99×10^{-6}	-0.009	0.028	0.760
Glutamate	rs12931566	16:69200931	A/T	-0.051	0.024	3.99×10^{-6}	-0.050	0.033	0.124
Glutamate	rs3775330	4:30728284	A/G	0.064	0.021	4.24×10^{-6}	-0.039	0.034	0.251
Glutamate	rs10041854	5:141464898	C/A	0.077	0.023	4.44×10^{-6}	-0.012	0.031	0.699
Glutamate	rs880626	15:60983429	G/A	0.049	0.017	4.55×10^{-6}	-0.035	0.031	0.261
Glutamate	rs10747028	9:80813104	C/A	0.053	0.014	4.81×10^{-6}	0.002	0.020	0.922
Serotonin	rs2742351	2:179541899	C/G	-0.024	0.005	3.23×10^{-7}	0.025	0.019	0.196
Serotonin	rs10516056	5:168468537	T/C	-0.022	0.005	6.99×10^{-7}	0.020	0.035	0.573
Serotonin	rs5764174	22:44033934	T/C	-0.019	0.004	9.61×10^{-7}	-0.021	0.020	0.309
Serotonin	rs8113773	19:30350139	A/G	0.016	0.003	1.12×10^{-6}	0.019	0.023	0.394
Serotonin	rs7868774	9:138016943	A/G	-0.019	0.004	1.78×10^{-6}	0.033	0.022	0.136
Serotonin	rs12646122	4:156246202	T/C	-0.05	0.01	1.80×10^{-6}	0.075	0.074	0.311
Serotonin	rs9614255	22:44056241	A/G	-0.019	0.004	1.93×10^{-6}	-0.019	0.020	0.358
Serotonin	rs825278	2:222561267	T/C	-0.091	0.019	2.06×10^{-6}	-0.004	0.076	0.962
Serotonin	rs739075	22:44034241	A/T	0.019	0.004	2.12×10^{-6}	0.021	0.020	0.313
Serotonin	rs5763947	22:44035303	A/G	-0.019	0.004	2.12×10^{-6}	-0.018	0.020	0.372
Serotonin	rs11103693	9:138016657	T/C	-0.019	0.004	3.09×10^{-6}	0.026	0.023	0.256
Serotonin	rs11103694	9:138016676	T/C	-0.019	0.004	3.10×10^{-6}	0.033	0.022	0.145
Serotonin	rs34724	19:30369010	T/G	-0.015	0.003	3.20×10^{-6}	-0.020	0.022	0.370
Serotonin	rs737788	22:44039285	T/C	-0.018	0.004	3.62×10^{-6}	-0.019	0.020	0.349
Serotonin	rs3753556	1:175162723	T/C	0.015	0.003	3.66×10^{-6}	-0.044	0.020	0.026

Serotonin	rs10881427	1:107172665	T/G	-0.015	0.003	3.69×10^{-6}	0.001	0.022	0.958
Serotonin	rs9614170	22:44061009	T/G	-0.018	0.004	3.87×10^{-6}	-0.021	0.020	0.294
Serotonin	rs10953038	7:90857574	T/C	0.022	0.005	4.12×10^{-6}	0.029	0.031	0.348
Serotonin	rs6466265	7:78160248	A/G	0.019	0.004	4.15×10^{-6}	-0.009	0.028	0.735
Serotonin	rs34722	19:30366802	A/C	0.015	0.003	4.36×10^{-6}	0.039	0.023	0.091
Serotonin	rs34720	19:30366245	A/G	0.015	0.003	4.40×10^{-6}	0.038	0.023	0.094
Serotonin	rs34725	19:30370237	A/G	-0.018	0.004	4.62×10^{-6}	-0.033	0.023	0.154
Serotonin	rs17039706	9:138016002	T/C	-0.019	0.004	4.74×10^{-6}	0.025	0.023	0.275

Note: For genetic variants (rs71640034, rs4947534, rs201393666, rs7979478, rs3753556) not available in the summary statistics of PD, proxied SNPs in linkage disequilibrium with them were utilized as bellows, rs4253311 (r2= 0.98), rs11238389 (r2= 0.99), rs190543502 (r2= 0.83), rs7970695 (r2= 0.99), and rs3753555 (r2= 1.00), respectively.

Abbreviations: EA, effect allele; OA, other allele; SNP, single nucleotide polymorphism.

Supplementary Table 4. Summary-level data associated with blood neurotransmitters and amyotrophic lateral sclerosis

Exposures	SNP	Position	EA/OA	Association with neurotransmitters			Association with amyotrophic lateral sclerosis		
				Beta	SE	*P*-value	Beta	SE	*P*-value
Glycine	rs715	2:211543055	C/T	0.444	0.006	2.23×10^{-308}	0.000	0.015	0.990
Glycine	rs9987289	8:9183358	A/G	0.124	0.01	1.74×10^{-49}	0.023	0.025	0.358
Glycine	rs9923732	16:81110903	A/G	0.119	0.011	1.22×10^{-41}	0.016	0.026	0.539
Glycine	rs17591030	9:6550024	C/T	0.08	0.006	1.88×10^{-40}	0.004	0.015	0.804
Glycine	rs4947534	7:56079094	C/T	0.072	0.007	7.12×10^{-34}	-0.015	0.016	0.325
Glycine	rs9862438	3:125910381	T/C	0.058	0.006	1.13×10^{-30}	-0.002	0.014	0.885
Glycine	rs4646961	1:76217169	A/G	0.048	0.006	8.41×10^{-19}	-0.015	0.015	0.314
Glycine	rs2545801	5:176841339	C/T	0.042	0.007	7.23×10^{-14}	-0.003	0.017	0.874
Glycine	rs561931	1:120254506	G/A	0.033	0.006	7.57×10^{-14}	-0.008	0.014	0.576
Glycine	rs12297321	12:47109387	T/C	0.048	0.008	7.41×10^{-13}	0.004	0.019	0.851
Glycine	rs10740134	10:65315433	T/C	0.038	0.006	1.18×10^{-12}	0.005	0.014	0.730
Glycine	rs8078686	17:45735706	C/T	0.035	0.006	3.66×10^{-11}	0.039	0.014	0.005
Glycine	rs543159	6:160776017	A/C	0.035	0.006	4.20×10^{-10}	-0.012	0.014	0.393
Glycine	rs71640034	4:187161048	A/G	0.034	0.006	5.57×10^{-10}	0.012	0.014	0.395
Glycine	rs10900807	5:131757480	G/C	0.036	0.007	1.26×10^{-9}	0.002	0.017	0.890
Glycine	rs10184004	2:165508389	T/C	0.036	0.006	1.53×10^{-9}	0.006	0.014	0.664
Glycine	rs2280195	15:58467095	A/G	0.028	0.006	3.15×10^{-9}	-0.005	0.014	0.744
Glycine	rs273510	19:18223350	A/G	0.034	0.006	3.57×10^{-9}	-0.007	0.015	0.626
Glycine	rs2638314	12:56866334	A/T	0.042	0.007	1.52×10^{-8}	0.015	0.017	0.389
Glycine	rs201393666	15:43677979	A/C	0.097	0.017	2.64×10^{-8}	-0.016	0.038	0.670
Glycine	rs9514191	13:104520138	C/G	0.034	0.006	3.10×10^{-8}	-0.038	0.015	0.012
Glycine	rs3105793	5:90226061	A/G	0.028	0.006	4.04×10^{-8}	0.003	0.015	0.821
Glycine	rs156380	5:53378450	C/T	0.031	0.007	4.50×10^{-8}	0.004	0.017	0.804
Glutamate	rs7979478	12:121420263	A/G	-0.018	0.014	1.52×10^{-10}	0.021	0.014	0.129
Glutamate	rs1183910	12:121420807	G/A	0.016	0.015	8.58×10^{-9}	-0.006	0.015	0.670
Glutamate	rs1169312	12:121441461	G/T	0.008	0.014	2.37×10^{-7}	-0.024	0.014	0.091
Glutamate	rs10842676	12:26261394	A/G	-0.04	0.014	2.91×10^{-7}	0.002	0.014	0.886

Glutamate	rs41356552	15:60957248	A/G	0.056	0.017	3.59×10^{-7}	0.009	0.017	0.594
Glutamate	rs339998	15:60947763	C/T	0.056	0.015	3.77×10^{-7}	-0.007	0.014	0.612
Glutamate	rs1182933	12:121454622	C/T	0.011	0.015	5.94×10^{-7}	-0.015	0.015	0.296
Glutamate	rs2650000	12:121388962	A/C	-0.004	0.015	1.52×10^{-6}	0.017	0.014	0.231
Glutamate	rs7110183	11:72551035	C/T	0.061	0.017	1.54×10^{-6}	0.015	0.017	0.355
Glutamate	rs5760492	22:24995202	G/A	-0.023	0.015	1.57×10^{-6}	0.006	0.015	0.704
Glutamate	rs10805985	6:74606851	G/A	0.046	0.015	2.09×10^{-6}	0.017	0.015	0.274
Glutamate	rs12301299	12:991710	T/C	-0.034	0.019	2.12×10^{-6}	-0.036	0.019	0.054
Glutamate	rs10774579	12:121405210	T/C	-0.021	0.014	2.30×10^{-6}	0.039	0.014	0.004
Glutamate	rs4895778	6:149436652	G/A	0.041	0.018	2.40×10^{-6}	0.026	0.018	0.142
Glutamate	rs12591786	15:60902512	C/T	0.048	0.02	2.66×10^{-6}	-0.001	0.019	0.961
Glutamate	rs3816411	3:13677751	T/C	-0.036	0.015	3.58×10^{-6}	-0.010	0.014	0.485
Glutamate	rs7956891	12:3475998	G/A	0.023	0.017	3.99×10^{-6}	-0.022	0.017	0.199
Glutamate	rs12931566	16:69200931	A/T	-0.051	0.024	3.99×10^{-6}	-0.007	0.023	0.769
Glutamate	rs3775330	4:30728284	A/G	0.064	0.021	4.24×10^{-6}	0.014	0.020	0.499
Glutamate	rs10041854	5:141464898	C/A	0.077	0.023	4.44×10^{-6}	0.008	0.022	0.711
Glutamate	rs712752	3:7024127	A/G	-0.048	0.017	4.52×10^{-6}	0.028	0.016	0.072
Glutamate	rs880626	15:60983429	G/A	0.049	0.017	4.55×10^{-6}	-0.005	0.016	0.751
Glutamate	rs10747028	9:80813104	C/A	0.053	0.014	4.81×10^{-6}	-0.005	0.014	0.726
Serotonin	rs2742351	2:179541899	C/G	-0.024	0.005	3.23×10^{-7}	-0.008	0.015	0.619
Serotonin	rs10516056	5:168468537	T/C	-0.022	0.005	6.99×10^{-7}	0.001	0.017	0.945
Serotonin	rs5764174	22:44033934	T/C	-0.019	0.004	9.61×10^{-7}	0.020	0.016	0.206
Serotonin	rs8113773	19:30350139	A/G	0.016	0.003	1.12×10^{-6}	0.006	0.014	0.686
Serotonin	rs7868774	9:138016943	A/G	-0.019	0.004	1.78×10^{-6}	0.002	0.015	0.902
Serotonin	rs12646122	4:156246202	T/C	-0.05	0.01	1.80×10^{-6}	0.033	0.048	0.491
Serotonin	rs9614255	22:44056241	A/G	-0.019	0.004	1.93×10^{-6}	0.023	0.016	0.154
Serotonin	rs825278	2:222561267	T/C	-0.091	0.019	2.06×10^{-6}	-0.021	0.090	0.817
Serotonin	rs739075	22:44034241	A/T	0.019	0.004	2.12×10^{-6}	-0.019	0.016	0.221
Serotonin	rs5763947	22:44035303	A/G	-0.019	0.004	2.12×10^{-6}	0.018	0.016	0.253
Serotonin	rs11103693	9:138016657	T/C	-0.019	0.004	3.09×10^{-6}	0.006	0.016	0.722
Serotonin	rs11103694	9:138016676	T/C	-0.019	0.004	3.10×10^{-6}	0.004	0.015	0.806
Serotonin	rs34724	19:30369010	T/G	-0.015	0.003	3.20×10^{-6}	-0.004	0.014	0.762

Serotonin	rs737788	22:44039285	T/C	-0.018	0.004	3.62×10^{-6}	0.020	0.016	0.206
Serotonin	rs3753556	1:175162723	T/C	0.015	0.003	3.66×10^{-6}	0.005	0.014	0.717
Serotonin	rs10881427	1:107172665	T/G	-0.015	0.003	3.69×10^{-6}	-0.007	0.014	0.628
Serotonin	rs9614170	22:44061009	T/G	-0.018	0.004	3.87×10^{-6}	0.019	0.016	0.225
Serotonin	rs10953038	7:90857574	T/C	0.022	0.005	4.12×10^{-6}	-0.010	0.019	0.608
Serotonin	rs6466265	7:78160248	A/G	0.019	0.004	4.15×10^{-6}	0.013	0.017	0.449
Serotonin	rs34722	19:30366802	A/C	0.015	0.003	4.36×10^{-6}	0.002	0.014	0.894
Serotonin	rs34720	19:30366245	A/G	0.015	0.003	4.40×10^{-6}	0.002	0.014	0.904
Serotonin	rs34725	19:30370237	A/G	-0.018	0.004	4.62×10^{-6}	-0.005	0.014	0.719
Serotonin	rs17039706	9:138016002	T/C	-0.019	0.004	4.74×10^{-6}	0.010	0.016	0.530

Abbreviations: EA, effect allele; OA, other allele; SNP, single nucleotide polymorphism.

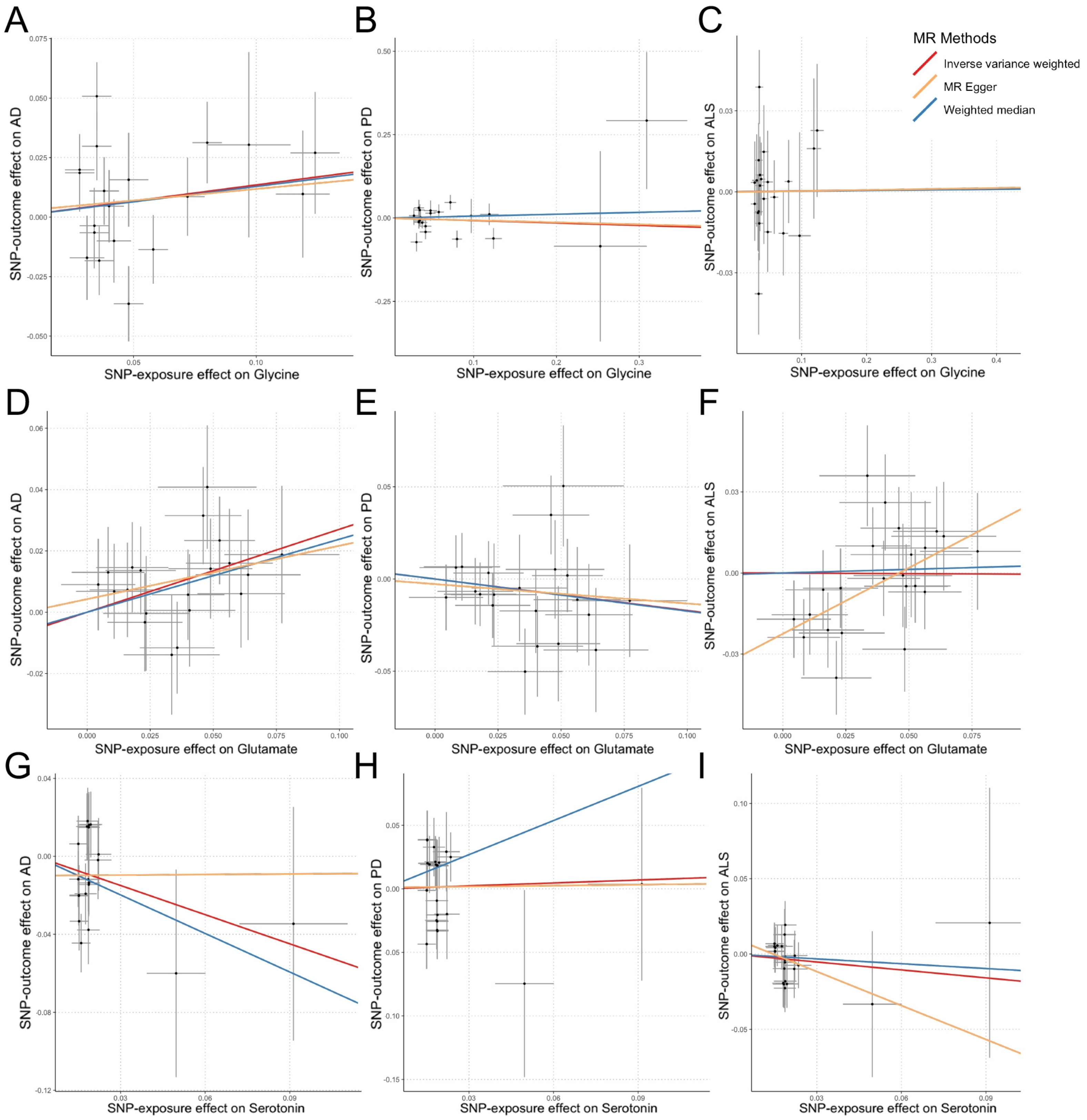
A
B
C
MR Methods
Inverse variance weighted
MR Egger
Weighted median
SNP-outcome effect on AD
SNP-outcome effect on PD
SNP-outcome effect on ALS
SNP-exposure effect on Glycine
D
E
F
SNP-exposure effect on Glutamate
G
H
I
SNP-exposure effect on Serotonin

Supplementary Figure 1. Scatter plots in Mendelian randomization analyses of circulating glycine, glutamate and serotonin on three neurodegenerative diseases. Overall causal estimates given by three MR methods, inverse-variance weighted, weighted median and MR-Egger were visualized using fitted lines with different colors. Instrumental SNPs were delineated with black solid points, with crossed horizontal and vertical gray lines depicting effect sizes for SNP-associations with neurotransmitters and neurodegenerative diseases, respectively. AD, Alzheimer's disease; ALS, amyotrophic lateral sclerosis; MR, Mendelian randomization; PD, Parkinson’s disease; SNP, single nucleotide polymorphism.

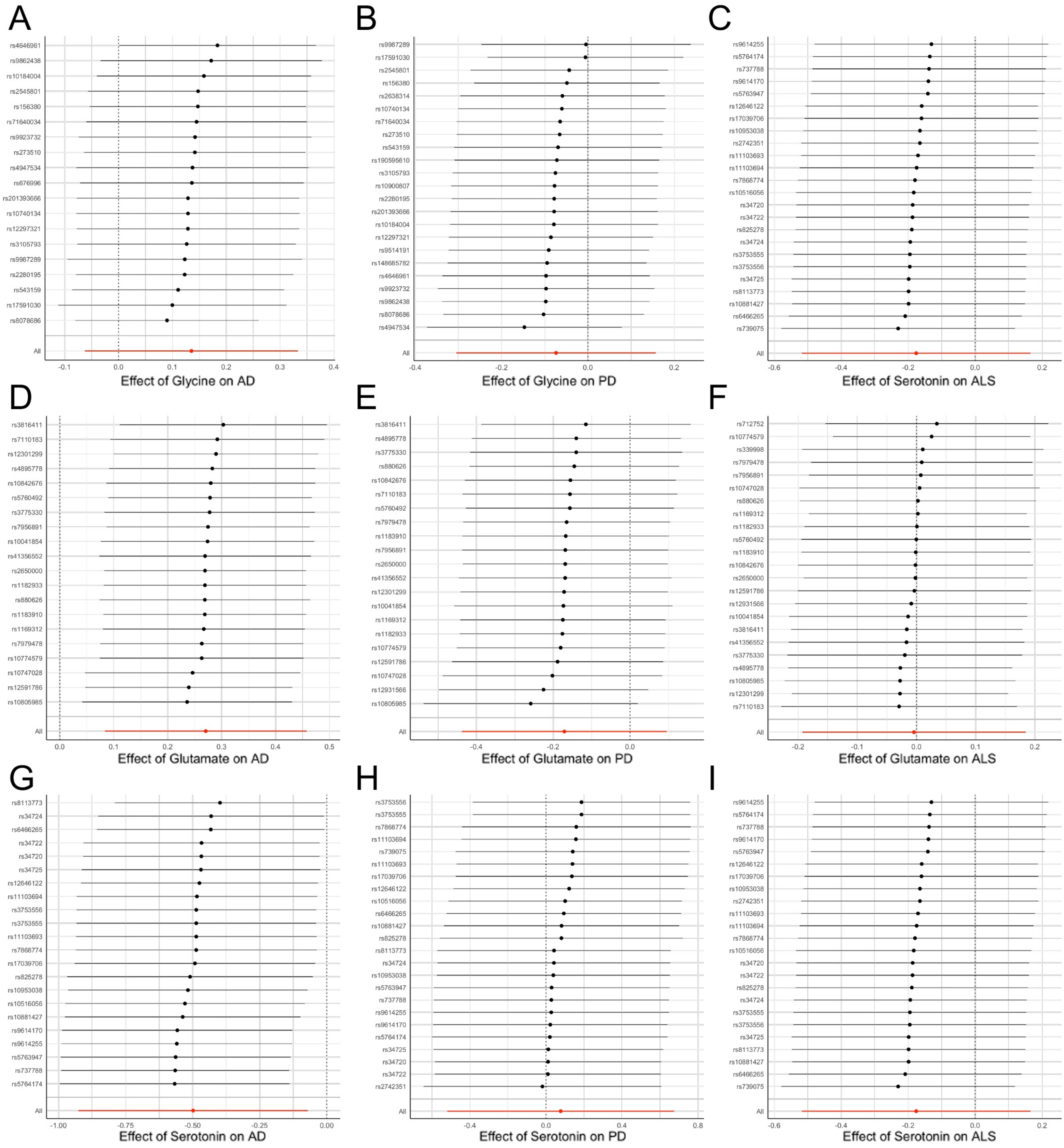
A
rs4646961
rs9862438
rs10184004
rs2545801
rs156380
rs71640034
rs9923732
rs273510
rs4947534
rs676996
rs201393666
rs10740134
rs12297321
rs3105793
rs9987289
rs2280195
rs543159
rs17591030
rs8078686
All
-0.1 0.0 0.1 0.2 0.3 0.4
Effect of Glycine on AD
B
rs9987289
rs17591030
rs2545801
rs156380
rs2638314
rs10740134
rs71640034
rs273510
rs543159
rs190595610
rs3105793
rs10900807
rs2280195
rs201393666
rs10184004
rs12297321
rs9514191
rs148685782
rs4646961
rs9923732
rs9862438
rs8078686
rs4947534
All
-0.4 -0.2 0.0 0.2
Effect of Glycine on PD
C
rs9614255
rs5764174
rs737788
rs9614170
rs5763947
rs12646122
rs17039706
rs10953038
rs2742351
rs11103693
rs11103694
rs7868774
rs10516056
rs34720
rs34722
rs825278
rs34724
rs3753555
rs3753556
rs34725
rs8113773
rs10881427
rs6466265
rs739075
All
-0.6 -0.4 -0.2 0.0 0.2
Effect of Serotonin on ALS
D
rs3816411
rs7110183
rs12301299
rs4895778
rs10842676
rs5760492
rs3775330
rs7956891
rs10041854
rs41356552
rs2650000
rs1182933
rs880626
rs1183910
rs1169312
rs7979478
rs10774579
rs10747028
rs12591786
rs10805985
All
0.0 0.1 0.2 0.3 0.4 0.5
Effect of Glutamate on AD
E
rs3816411
rs4895778
rs3775330
rs880626
rs10842676
rs7110183
rs5760492
rs7979478
rs1183910
rs7956891
rs2650000
rs41356552
rs12301299
rs10041854
rs1169312
rs1182933
rs10774579
rs12591786
rs10747028
rs12931566
rs10805985
All
-0.4 -0.2 0.0
Effect of Glutamate on PD
F
rs712752
rs10774579
rs339998
rs7979478
rs7956891
rs10747028
rs880626
rs1169312
rs1182933
rs5760492
rs1183910
rs10842676
rs2650000
rs12591786
rs12931566
rs10041854
rs3816411
rs41356552
rs3775330
rs4895778
rs10805985
rs12301299
rs7110183
All
-0.2 -0.1 0.0 0.1 0.2
Effect of Glutamate on ALS
G
rs8113773
rs34724
rs6466265
rs34722
rs34720
rs34725
rs12646122
rs11103694
rs3753556
rs3753555
rs11103693
rs7868774
rs17039706
rs825278
rs10953038
rs10516056
rs10881427
rs9614170
rs9614255
rs5763947
rs737788
rs5764174
All
-1.00 -0.75 -0.50 -0.25 0.00
Effect of Serotonin on AD
H
rs3753556
rs3753555
rs7868774
rs11103694
rs739075
rs11103693
rs17039706
rs12646122
rs10516056
rs6466265
rs10881427
rs825278
rs8113773
rs34724
rs10953038
rs5763947
rs737788
rs9614255
rs9614170
rs5764174
rs34725
rs34720
rs34722
rs2742351
All
-0.4 0.0 0.4 0.8
Effect of Serotonin on PD
I
rs9614255
rs5764174
rs737788
rs9614170
rs5763947
rs12646122
rs17039706
rs10953038
rs2742351
rs11103693
rs11103694
rs7868774
rs10516056
rs34720
rs34722
rs825278
rs34724
rs3753555
rs3753556
rs34725
rs8113773
rs10881427
rs6466265
rs739075
All
-0.6 -0.4 -0.2 0.0 0.2
Effect of Serotonin on ALS

Supplementary Figure 2. Leave-one-out plots in Mendelian randomization analyses of circulating glycine, glutamate and serotonin on three neurodegenerative diseases. Leave-one-out plots depicted overall effect estimates given by the inverse-variance weighted MR with each individual SNP excluded out of the instrumental variable set in turn. Causal effect sizes underwent natural logarithm transformation, and point estimates were shown with solid circles, whereas 95% confidence intervals were illustrated with horizontal lines through them. Vertical dashed lines represented null effects, and the left and right parts indicated the protective and deleterious effects of circulating neurotransmitters on neurodegenerative diseases, respectively. There was no evidence for the existence of heterogenous variables, which would otherwise disproportionately drove the overall effects. AD, Alzheimer's disease; ALS, amyotrophic lateral sclerosis; MR, Mendelian randomization; PD, Parkinson's disease; SNP, single nucleotide polymorphism.
